# Quality of measurement instruments assessing Planetary and One Health Literacy related constructs: a systematic review

**DOI:** 10.1186/s12955-026-02652-3

**Published:** 2026-10-16

**Authors:** Lydia Reismann, Jennifer Kallenbach, Christian Apfelbacher, Theresa Donhauser, Claudia Hasenpusch, Jana Hinneburg, Anke Steckelberg, Carmen Jochem

**Affiliations:** 1 Chair of Planetary & Public Health, University of Bayreuth, Universitätsstraße 30, 95447 Bayreuth, Germany; 2 Department of Education, Faculty of Human Sciences, MSH Medical School Hamburg, Am Kaiserkai 1, 20457 Hamburg, Germany; 3 Institute of Social Medicine and Health Systems Research, Medical Faculty, Otto von Guericke University Magdeburg, Leipziger Str. 44, 39120 Magdeburg, Germany; 4 Institute of Health, Midwifery and Nursing Science, Medical Faculty of Martin Luther University Halle-Wittenberg, University Medicine Halle, Magdeburger Str. 8, 06112 Halle (Saale), Germany

**Keywords:** Instrument, Health literacy, Ecology, Assessment, Scale development, Environmental health, Transformative, Sustainability, Health promotion, One Health

## Abstract

**Background:**

Amid accelerating anthropogenic climate and ecological crises, the expansion of health literacy as a comprehensive and multidimensional concept addressing upstream determinants of health has become relevant. A critical gap persists in measurement research to identify validated instruments for health literacy within the environment-health nexus.

**Objective:**

To conduct a systematic review of the quality of existing instruments that measure Planetary and One Health Literacy or related constructs according to the COnsensus-based Standards for the selection of health Measurement INstruments (COSMIN) methodology.

**Methods:**

A systematic literature search was performed in Medline, Embase, Web of Science, CINAHL, APA PsychInfo, APA PsycArticles, PSYNDEX, and Greenline to identify development and/or validation studies of suitable instruments. We assessed the risk of bias of each study, systematically evaluated the psychometric properties of instruments, and graded the certainty of evidence to inform recommendations for instrument use and further development.

**Results:**

Out of 192 screened records, we identified four studies employing three instruments: Climate Change Health Literacy Scale (CCHLS), Climate Change and Infectious Disease Literacy Scale, and Sustainable Food Literacy Scale (SFLS). The quality of evidence for the development and content validity in all instruments was rated low or very low. Of all scales the SFLS demonstrated the best results in the assessed measurement properties supported by very good quality of evidence in its original, while its Turkish adaptation had low methodological quality in its assessed measurement properties and insufficient reliability.

**Conclusion:**

The included studies emphasised quantitative validation over robust instrument development and transparency in their reporting, highlighting the need for clearly defining constructs and involving target populations in the design processes. While existing measurement instruments primarily assess knowledge, attitudes, and behaviour, the lack of attention to the health literacy’s construct calls for stronger integration between health literacy, Planetary Health, and One Health research.

**Supplementary Information:**

The online version contains supplementary material available at https://doi.org/10.1186/s12955-026-02652-3.

## Introduction

Health literacy is widely recognised as a critical determinant of public health outcomes, particularly in advancing health equity [1, 2]. Drawing upon the plurality of existing definitions, an integrated model of health literacy encompasses a process-oriented conceptualization of health literacy, including the skills “*to acquire the necessary information*,* understanding this information*,* critically analyzing and appraising it*,* and acting independently to engage in actions overcoming personal*,* structural*,* social and economical barriers to health.*” [3]. The impacts of climate change, pollution, and biodiversity loss in their interdependence with health are exacerbating disparities both within countries and between them [4, 5]. Recent developments suggest an expansion of health literacy as a comprehensive and multidimensional concept addressing upstream determinants of health in their connection with ecological stability, social equity, and human well-being: Planetary Health Literacy and One Health Literacy [6–9].

Planetary Health Literacy, as defined in the conceptual model by Jochem et al. [6] as “*the knowledge and competencies of accessing*,* understanding*,* appraising*,* and applying information in order to make judgements and take decisions regarding planetary health*,* across societies and for health-promoting*,* sustainable*,* and transformative actions”*, provides a comprehensive and holistic approach similar to One Health Literacy. One Health Literacy can be defined as “*the knowledge*,* motivation and competencies to access*,* understand*,* appraise and apply all relevant information that are related to One Health […] to sustainably balance the health and quality of life of us humans*,* the animals and the environment”* [9]. Both concepts are rooted in and mutually reinforcing existing approaches like EcoHealth Literacy, Climate Change and Health Literacy, or Climate-specific Health Literacy [6], with the shared vision of supporting informed personal and collective health decision-making in regard to the environment-health nexus [10].

Empirical evidence from the literature indicates a correlation between enhanced health literacy and the uptake of healthier behaviours across different age groups [11, 12], suggesting a similar potential for planetary health behaviour.

Despite the importance of strengthening health literacy, a significant gap persists in the attention given to the measurement and assessment research, and how this can inform the design, implementation, and evaluation of effective, equity-focused interventions [2, 13]. Similarly, an instrument that can accurately measure Planetary Health Literacy or One Health Literacy is critical to design targeted Planetary and One Health interventions for individuals and communities as well as enable population-level analyses, including epidemiological surveillance, trend monitoring, and cross-regional or cross-national comparisons. In light of the similarities of concepts and approaches [14, 15], it is essential to undertake a comprehensive review of the existing measurement instruments for constructs related to Planetary and One Health Literacy. To our knowledge to date, no comprehensive assessment of availability and quality of instruments designed for related concepts of Planetary and One Health Literacy has been conducted. Only one systematic review has been published on a similar topic, which focused exclusively on instruments measuring nurses’ knowledge and awareness of climate change and did not include a clear operationalisation of health literacy [16].

Thus, the overarching aim of this study is to conduct a systematic review of the quality of existing instruments that measure Planetary and One Health Literacy or related constructs. More specifically, the study aims to (i) identify relevant instruments, (ii) evaluate the methodological quality of the development and/or validation studies, and to (iii) assess the certainty of evidence; (iv) derive recommendations for the use of the identified Planetary and One Health Literacy instruments in future research.

## Methods

This study is reported in accordance with the COnsensus-based Standards for the selection of health Measurement INstruments (COSMIN) guidelines and manual [17, 18] and the Preferred Reporting Items for Systematic reviews and Meta-Analyses (PRISMA)-COSMIN Reporting Guidelines for studies on measurement properties [19]. Although the COSMIN guidelines were initially created for Patient-Reported Outcome Measures (PROMs), they can be used to evaluate non-PROMs instruments [18]. The use of non-PROM instruments has been explored in prior health literacy research [16, 20]. The study adhered to the study protocol which was preregistered with PROSPERO (CRD420251105706) and OSF Registries (https://osf.io/735gn) prior to data extraction.

During manuscript preparation, the authors used Qwen3-30B [21] for language editing and spell-checking; after using this tool, the authors reviewed and edited the content as needed and take full responsibility for the content of the published article.

### Search strategy

The search strategy was collaboratively developed by all authors following COSMIN step 2 (eligibility criteria) and 3 (literature search) [17], including pilot-testing in June 2025. We used seed references, which were selected based on the included constructs, to benchmark our strategy. A librarian consultation on July 7, 2025 ensured clarity in our scope, maintaining the original search strategy without refinement and attributing the small numbers of records to the nascent stage of the research field.

The search string comprises four thematic blocks: (a) ‘health’; (b) ‘literacy’ or ‘competency’ related to (c) ecological aspects (‘ecohealth’, ‘climate change and health’, ‘one health’, or ‘planetary health’, ‘biodiversity’); and (d) adapted methodological terms suggested by the COSMIN guidelines [22]. Proximity operator ‘NEAR/n’ is used between b) and c). The full search string is provided in the Additional file 1.

The systematic literature search was performed on July 16, 2025 in the bibliographic databases MEDLINE (via Ovid), EMBASE (via Ovid), Web of Science Core Collection, CINAHL (EBSCO), APA PsychInfo (EBSCO), APA PsycArticles (EBSCO), PSYNDEX (EBSCO), and Greenline (EBSCO). As recommended for the research scope [23], a comprehensive hand search of reference lists, Google scholar (first 100 records), and six databases specific for outcome measurement instruments (see Additional file 2), was conducted to find additional instruments and literature. If additional instruments were identified during hand searching, all databases were re-searched using the names of the newly found instruments.

### Eligibility criteria

The eligibility criteria covered studies that describe the development and validation of instruments. Both qualitative and quantitative studies were eligible, with no restrictions on date, or language. Unpublished, non-peer-reviewed data of instruments were excluded from eligibility. Criteria for inclusion/exclusion are summarised in Table 1.

**Table 1:** Inclusion and exclusion criteria for the systematic review

	Inclusion criteria	Exclusion criteria
Construct of interest	Health Literacy and related competencies, skills, or domains (access, understand, appraise, apply)	All other constructs
Concept of interest	Literacy and health literacy in the context of global environmental change, climate crisis, ecological sustainability from a health perspective	Health literacy without global ecological, planetary, or climate-related aspects (e.g. in a local context of specific environmental hazards) OR Environmental related competencies or literacy concepts without health aspects
Population	This review does not have any restrictions on population.	
Instrument type	All types of instruments / assessment tools measuring the above-mentioned concept and construct	All other types of instruments
Study Design	Development or validation studies for all types of measurement instruments	All other study designs
Language	Available in or translatable to English	All other languages

Expecting heterogeneity of constructs and concepts [14, 24, 25] all authors reached consensus through repeated discussion: for the purpose of this review, we adopted a narrow understanding that excludes vague and broad synonyms of the literacy construct with a precise focus on health literacy measurement and not solely addressing *knowledge*,* skills*, or *attitudes.* For the conceptual boundaries, the definitions outlined in the introductory Section [6, 9]. were consistently applied and a concept is considered *related to Planetary and One Health Literacy* if it explicitly acknowledges and integrates the interdependence among human, animal, and ecological health. While not necessarily encompassing all three domains, a study must demonstrate a clear conceptual focus on the dynamic, reciprocal relationships within an integrated, systems-based perspective to reflect the core principle of interconnectedness of Planetary and One Health Literacy [10].

### Study selection

Following deduplication of the records in Citavi 7 [26], screening and study selecting process was conducted in Rayyan, an online-tool facilitating collaboration among the research team [27]. Prior to the formal screening process, a consistency check with ten studies was performed among all authors participating in screening (LR, JK, CJ, JH, AS) to pilot the eligibility criteria and conceptual consensus. Title and abstracts of all identified publications were independently screened by two researchers out of a team of five reviewers against predefined eligibility criteria. When the eligibility of a publication was unclear during this initial screening phase, the full text of the article was reviewed. If the fulltext was unavailable, authors were contacted and asked to provide their publication. For articles considered eligible at the initial stage, full-text screening was conducted also by two reviewers (LR, JK), ensuring 100% overlap between them. Any disagreements or uncertainties were resolved by a third reviewer. Reasons for exclusion during the full-text screening phase were thoroughly documented.

### Data extraction

After the full-text screening, data extraction (COSMIN step 5) was performed using a pre-defined excel-based data extraction form provided by COSMIN (*review management file*), including instrument characteristics, feasibility, interpretability and study populations [18]. COSMIN standards demand to analyse each subscale independently. A single reviewer (LR) conducted the data extraction (instrument characteristics, COSMIN step 5) with an evaluation by a second reviewer (JK). The full review management file is available in the Additional file 3. For the clarification of unclear or missing information, the corresponding authors of all studies were contacted to provide responses via e-mail within a two-week deadline.

### Assessment of the methodological quality of included studies

The methodological quality of included studies was evaluated using the COSMIN Risk of Bias checklist [28, 29]; the COSMIN Risk of Bias boxes are shown in Table 2. The quality of studies was rated independently by two reviewers (LR, JK) on a four-point scale (“very good”, “adequate”, “doubtful”, or “inadequate”) with a total score based on the lowest rating (“worst score counts”) [29]. Any disagreements were resolved by discussion until consensus was reached.

**Table 2:** Boxes of the COSMIN Risk of Bias checklist [28] and definitions of measurement properties from COSMIN standards [17]

Boxes of the COSMIN Risk of Bias checklist		Definition of measurement properties
**Content validity**		
Box 1	Instrument development	-
Box 2	Content validity	The degree to which the content of an instrument is an adequate reflection of the construct to be measured
**Internal structure**		
Box 3	Structural validity	The degree to which the scores of an instrument are an adequate reflection of the dimensionality of the construct to be measured
Box 4	Internal consistency	The degree of interrelatedness among the items
Box 5	Cross-cultural validity/measurement invariance	The degree to which the performance of the items on a translated or culturally adapted instrument are an adequate reflection of the performance of the items of the original version of the instrument
**Remaining measurement properties**		
Box 6	Reliability	The proportion of the total variance in the measurements which is due to ‘true’ differences between patients
Box 7	Measurement error	The systematic and random error of a score that is not attributed to true changes in the construct to be measured
Box 8	Criterion validity	The degree to which the scores of an instrument are an adequate reflection of a gold standard
Box 9	Hypotheses testing for construct validity	The degree to which the scores of an instrument are consistent with hypotheses (e.g. with regard to internal relationships, relationships to scores of other instruments, or differences between relevant groups) based on the assumption that the instrument validly measures the construct to be measured
Box 10	Responsiveness	The ability of an instrument to detect change over time in the construct to be measured

### Evaluation of measurement properties and grading

A total of nine measurement properties were evaluated according to COSMIN step 6 [18] with their definitions presented in Table 2. The quality of measurement properties was assessed against updated criteria recommended by the COSMIN group [18] shown in Table 3. Content validity was rated independently by two reviewers (LR, JK). The other measurement properties were assessed by a single reviewer (LR) as sufficient (+), insufficient (-), or indeterminate (?), with a second reviewer (JK) evaluating all data.

**Table 3:** Criteria for good measurement properties

Measurement property	Rating	Criteria
Content validity	+	Included items are relevant for the construct, target population, and context of use, and response options and recall period are appropriate AND No key concepts are missing AND PROM items and response options are appropriately worded and PROM instructions, items and response options understood by the population of interest as intended
	?	Not enough information reported
	-	Included items are not relevant for the construct or target population OR Key concepts are missing OR PROM items and response options are not appropriately worded or not understood by the population of interest as intended
Structural validity	+	**CTT**: EFA/PCA: factor loadings of each item on its factor ≥ 0.30 AND Maximum 10% of the items have factor loadings ≥ 0.30 on multiple factors AND Explained variance ≥ 50% and structure is in line with the theory about the construct to be measured OR results on scree plot or Kaiser criterion (Eigenvalues > 1) are in line with the theory about the construct to be measured CFA: CFI or TLI or comparable measure > 0.95 OR RMSEA < 0.06 OR SRMR < 0.08 **IRT/Rasch**: No violation of unidimensionality: CFI or TLI or comparable measure > 0.95 OR RMSEA < 0.06 OR SRMR < 0.08 AND No violation of local independence: residual correlations among the items after controlling for dominant factor < 0.20 OR Q3s < 0.37 AND No violation of monotonicity: adequate looking graphs OR item scalability > 0.30 AND Adequate model fit: IRT: χ2 > 0.01 Rasch: infit and outfit mean squares ≥ 0.5 and ≤ 1.5 OR Z-standardized values >-2 and < 2
	?	Not enough information reported
	-	Criteria for ‘+’ not met
Internal consistency	+	At least low evidence for sufficient unidimensionality AND Cronbach’s alpha ≥ 0.70
	?	Criteria for “at least low evidence for sufficient unidimensionality” not met OR Evidence for insufficient unidimensionality OR Not enough information reported
	-	At least low evidence for sufficient structural validity and Cronbach’s alpha(s) < 0.70
Reliability	+	ICC or (weighted) kappa or Pearson/Spearman correlation ≥ 0.70
	?	Not enough information reported
	-	ICC or (weighted) kappa or Pearson/Spearman correlation < 0.70
Measurement error	+	SDC or LoA < MIC
	?	MIC not defined OR not enough information reported
	-	SDC or LoA > MIC
Hypotheses testing for construct validity	+	≥ 75% of the results is in accordance with predefined hypotheses
	?	No relevant results were found
	-	≥ 75% of the results deviates from predefined hypotheses
Cross-cultural validity/measurement invariance	+	No important differences found between group factors (such as age, gender, language) in multiple group factor analysis OR no important DIF for group factors (McFadden’s R^2^ < 0.02)
	?	Not enough information reported
	-	Important differences between group factors OR DIF was found
Criterion validity	+	Correlation with gold standard ≥ 0.70 OR AUC ≥ 0.70
	?	Not all information for ‘+’ reported
	-	Correlation with gold standard < 0.70 OR AUC < 0.70
Responsiveness	+	The result is in accordance with the hypothesis OR AUC ≥ 0.70
	?	No relevant results were found
	-	≥ 75% of the results deviates from predefined hypotheses OR AUC < 0.70

COSMIN criteria according to Mokkink et al. [18]; “+” = sufficient, “-“ = insufficient, “?” = indeterminate; AUC area under the curve, CFA confirmatory factor analysis, CFI comparative fit index, CTT classical test theory, DIF differential item functioning, EFA exploratory factor analysis, ICC intraclass correlation coefficient, IRT Item response theory, LoA limits of agreement, MIC minimal important change, RMSEA root mean square error of approximation, SEM standard error of measurement, SDC smallest detectable change, SRMR standardised root mean residuals, TLI Tucker-Lewis index, PROM Patient-Reported Outcome Measure

In accordance with the COSMIN guidelines, the quality of evidence was graded using a modified Grading of Recommendations Assessment, Development, and Evaluation (GRADE) approach [17]. The evidence grading per measurement property per instrument began by assuming high-quality evidence but was downgraded (quality of evidence as moderate, low, or very low) if any of the following was present: risk of bias, inconsistency, indirectness, or imprecision [18]. The quality of evidence was not graded if an overall rating was indeterminate.

## Results

**Fig. 1:**
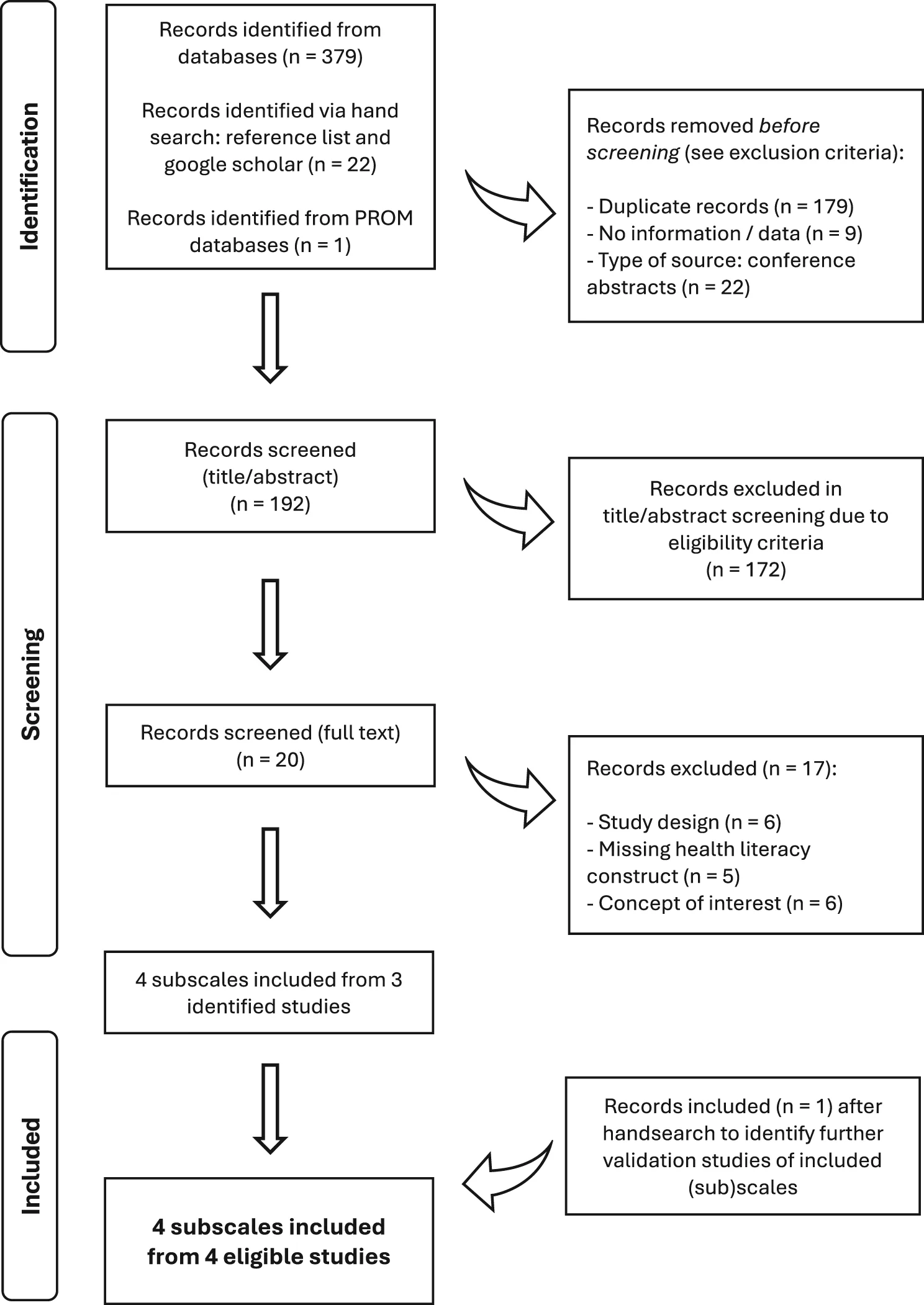
Data collection flow chart detailing the screening process for the COSMIN-based systematic review; adapted from PRISMA flow chart

The database and hand search resulted in 192 unique abstracts, of which 20 were assessed in a full-text screening as shown in our flow chart (Fig. 1). After the full-text screening, three articles met the inclusion criteria providing four unique subscales to be included in this review. The hand search for all scales identified one further validation study, resulting in four included studies.

While multiple studies initially appeared potentially eligible during full-text screening, they were formally excluded based on rigorous inclusion criteria. A comprehensive documentation of excluded studies with the rationale for exclusion is given in the Additional file 4; this also informed the identification of research gaps. While formally excluded, detailed assessment of these near-eligible studies identified five articles with preliminary scales which were excluded due to missing validation or development study design [8, 30–33], six articles with a similar concept in the environment-health nexus but lacking a health literacy construct [34–39], and six studies describing a different conceptual ground (e.g. environmental awareness without a clear health link) [40–45].

### Characteristics of included instruments

The included scales measured related constructs within the broader domain of extended health literacy in the environmental-health nexus: the Climate Change Health Literacy Scale (CCHLS) [46], the Sustainable Food Literacy Scale (SFLS) [47], and two subscales from the Climate Change and Infectious Disease Literacy Scale [48]. The characteristics of each included scale are presented in Table 4.

**Table 4:** Characteristics of the included instruments

	Climate Change Health Literacy Scale (CCHLS)	Sustainable Food Literacy Scale (SFLS)	Climate Change and Infectious Disease Literacy Scale – Climate Subscale	Climate Change and Infectious Disease Literacy Scale – VBD Subscale
Developed by author (year)	Nayir et al. (2025)	Teng & Chih (2022)	Matlack et al. (2023)	Matlack et al. (2023)
Construct (outcome domain as developed)	Health literacy (knowledge, attitudes, behaviours) regarding climate change impacts on health	Sustainable Food Literacy (sustainable food knowledge, food skills, attitudes, action intent, and action strategies)	Literacy (knowledge, attitudes, behaviours) regarding climate change impacts on health	Literacy (knowledge, attitudes, behaviours) regarding vector-borne disease dynamics
Origin of the construct (theory, model, conceptual framework)	Unclear; no information provided	Unclear; construct created by the authors informed on prior research	Unclear; construct created by the authors informed by a prior environmental literacy scale found in a scoping review [15]	Unclear; construct created by the authors informed by a prior environmental literacy scale found in a scoping review [15]
Target population (as developed)	Turkish adults aged 18+	Taiwanese adults aged 18+	Women currently enrolled in the Caribbean Consortium for Research in Environmental and Occupational Health cohort in Suriname	Women currently enrolled in the Caribbean Consortium for Research in Environmental and Occupational Health cohort in Suriname
Mode of administration	Self-administered questionnaire (face-to-face)	Self-administered questionnaire (web-based)	Self-administered questionnaire (face-to-face)	Self-administered questionnaire (face-to-face)
Number of items	24	26	11	9
Factors/ Dimensions (after EFA/CFA)	4-dimensional: Health impact (11 items), monitoring (6 items), behavioural (2 items), prevention-support (5 items)	4-dimensional: Sustainable food knowledge (9 items), food skills (6 items), attitudes (4 items), action intent/strategies (merged, 7 items)	4-dimensional: General climate effects (3 items), heat effects (4 items), cooling/indoor behaviours (2 items), ocean warming (2 items)	4-dimensional: Disease transmission (3 items), temperature effects (2 items), viruses (2 items), water-holding containers (2 items)
Response options	5-point Likert scale (1 = strongly disagree to 5 = strongly agree)	7-point Likert scale (1 = strongly disagree to 7 = strongly agree)	5-point Likert scale (1 = strongly disagree to 5 = strongly agree)	5-point Likert scale (1 = strongly disagree to 5 = strongly agree; 1 = never to 5 = always)
Scoring algorithm	Higher scores indicate greater literacy	Higher scores indicate greater literacy	Higher scores indicate greater literacy	Higher scores indicate greater literacy
Score range	24–120	26–182	11–55	9–45
Original language	Turkish (available upon request by authors)	Chinese (available upon request by authors)	Dutch (not publicly accessible)	Dutch (not publicly accessible)
Available translations	English translation for research purposes	English translation for research purposes; adopted Turkish version [49]	English translation for research purposes	English translation for research purposes
Context of use (as developed)	Unclear; Individual-level assessment; educational intervention evaluation; policy recommendations.	Unclear; Sustainable diet education interventions; adult literacy evaluation (students, consumers, employees)	Unclear; Community engagement projects; preventive practice capacity building in vulnerable regions	Unclear; Community engagement projects; preventive practice capacity building in vulnerable regions
Measurement model	Reflective	Reflective	Formative reflective mixed	Formative reflective mixed

The CCHLS was developed “to assess the relationship between climate change and health at the individual level” and contains 24 items to self-assess knowledge, attitudes, and behaviours regarding the health impacts of climate change [46]. The SFLS was designed to self-assess the dimensions sustainable food knowledge, food skills, attitudes, and action intent/strategies related to sustainable health practices in adult populations [47]. It has a Turkish adaptation [49] with similar dimensions in a five-factor structure. The Climate Change and Infectious Disease Literacy Scale comprises two subscales measuring knowledge, attitudes, and behaviours regarding general climate change effects and vector-borne disease transmission dynamics in women from the Caribbean and South American regions [48].

As presented in Table 5, for all instruments, structural validity (with both exploratory and confirmatory factor analyses), content validity with professionals, and internal consistency (Cronbach’s alpha) were evaluated. For the SFLS, measurement invariance, reliability, and its Turkish adaptation were additionally assessed. Sample characteristics (Table 5) differed across studies. Age distributions were generally broad (spanning adults 18 + years).

**Table 5:** Study characteristics

Scale	Author (Year)	Sample (*n*)	Study Design	Measurement Properties Evaluated	Content validity (type of professionals)
Climate Change Health Literacy Scale (CCHLS)	Nayir et al. (2025)	*n* = 318 Turkish adults; pilot study *n* = 82	Cross-sectional	Content validity, structural validity (EFA, CFA), internal consistency	*n* = 14 (7 medical doctors, 3 public health experts, 2 scale development experts and 2 environmental engineers)
Sustainable Food Literacy Scale (SFLS)	Teng & Chih (2022)	EFA: *n* = 301; CFA: *n* = 667 Taiwanese adults	Cross-sectional	Content validity, structural validity (EFA, CFA), internal consistency, cross-cultural validity (measurement invariance by gender)	*n* = 7 (experts in food and nutrition literacy)
Sustainable Food Literacy Scale (SFLS): Turkish adoption	Kubilay & Yuksel (2023)	*n* = 533; Turkish adults; pilot study *n* = 50	Cross-sectional	Pilot study (comprehensibility), structural validity (EFA), internal consistency, reliability	Translation approved by 4 experts (NA)
Climate Change and Infectious Disease Literacy Scale - Climate	Matlack et al. (2023)	*n* = 301 (sample split: EFA: *n* = 180; CFA: *n* = 121); women in Suriname	Cross-sectional	Content validity, structural validity (EFA, CFA), internal consistency	*n* = 6 (NA)
Climate Change and Infectious Disease Literacy Scale - VBD	Matlack et al. (2023)	*n* = 301 (sample split: EFA: *n* = 180; CFA: *n* = 121); women in Suriname	Cross-sectional	Content validity, structural validity (EFA, CFA), internal consistency	*n* = 6 (NA)

EFA exploratory factor analysis; CFA confirmatory factor analysis; NA not applicable/no information available

### Assessing the development and content validity of the scales

All instruments were developed deductively by their authors drawing from existing literature, with the CCHLS and SFLS reported to be informed by non-systematic literature reviews [46, 47] and the concept definition of the Climate Change and Infectious Disease Literacy Scale by its prior scoping review [15, 48]. As no clear rationale for construct inclusion was reported and no theoretical models or conceptual frameworks were explicitly referenced or qualitatively developed as a concept elicitation study, the origin of construct was rated as unclear per COSMIN standards [17]. Similarly, limited information was provided regarding intended context of use, target population specificity, and item selection procedures across all instruments.

Content validity assessments for all instruments primarily followed expert panels. CCHLS used the Lawshe quantitative content validity method with 14 professionals rating item relevance leading to the exclusion of 22 out of 53 items. As COSMIN’s criteria for good content validity require that experts rate at least 85% of the items relevant for the construct [17], relevance was rated insufficient. The SFLS utilised a 7-expert panel assessing wording and construct alignment, yielding sufficient results; and the Climate Change and Infectious Disease Literacy Scale applied six experts using categorical ratings (“essential/useful/not needed”) with predefined 80%/67% agreement thresholds where more than 50% of the items did not meet the threshold and were retained [48], resulting in an insufficient relevance rating for both subscales according to the COSMIN’s criteria for good content validity [17].

Comprehensibility was mentioned in experts’ content validity assessments only for the SFLS in both the Taiwanese [47] and the Turkish [49] scale. Pilot testing on comprehensibility with the target group was limited to CCHLS, where adults provided face-to-face feedback on item clarity (*n* = 82) [46], and to the Turkish version of the SFLS (*n* = 50, no information about the setting) [49], both without documented interview guides, verbatim transcription, or analysis of response options/instructions, resulting in doubtful risk-of-bias ratings per COSMIN standards.

The evidence for the comprehensiveness of the scales was not provided, as no information was reported regarding the extent to which the items cover the construct of interest. Detailed content validity assessments are provided in Table 6, including reviewers’ ratings. The Turkish adoption of the SFLS [49] could not be assessed separately due to insufficient information. The reviewers rated the relevance of the items in three out of the four subscales as sufficient. The relevance of the climate subscale [48] was rated insufficient as it measured a different concept than the intersection of climate and health. The comprehensiveness across all instruments was rated insufficient by reviewers because systemic and justice perspectives as well as the health literacy domain of appraising were scarcely measured.

**Table 6:** Assessment of the development and content validity of the scales

Scale	Development study	Content validity by experts			Content validity by reviewers‘ rating			Target group	Overall rating	Quality of evidence
	Concept elicitation study / item development	Relevance	Comprehensiveness	Comprehensibility	Relevance	Comprehensiveness	Comprehensibility		+ / - / ? / ±	High, moderate, low, very low
Climate Change Health Literacy Scale (CCHLS)	Inadequate (?)	Doubtful (?)	NCA	NCA	?	?	+	Pilot study comprehensibility: Doubtful (+)	?	Very low
Sustainable Food Literacy Scale (SFLS)*	Inadequate (?)	Doubtful (?)	NCA	Doubtful (?)	+	+	±	*Pilot study comprehensibility: Doubtful (+)	±	Very low
Climate Change and Infectious Disease Literacy Scale - Climate	Inadequate (?)	Doubtful (-)	NCA	NCA	?	?	+	not involved	?	Very low
Climate Change and Infectious Disease Literacy Scale - VBD	Inadequate (?)	Doubtful (-)	NCA	NCA	?	?	+	not involved	?	Very low

Risk of Bias ratings: *very good*,* adequate*,* doubtful*,* inadequate*,* NCA* (not clearly assessed). Quality of measurement properties: *+ = sufficient; - = insufficient; ? = indeterminate; ± = inconsistent*. Summarized quality of evidence: *high*,* moderate*,* low*,* very low*

*The Turkish version of the SFLS was not assessed separately due to lack of information. The data in the SFLS assessment comes from the original study by Teng & Chih (2022), the only extracted information from Kubilay & Yuksel (2023) is the pilot study regarding target groups’ comprehensibility

### Assessment of the remaining measurement properties

In total, the development studies of the instruments [46–48] showed very good methodological quality on at least one measurement property. The summarised results from the risk of bias assessment, the quality of measurement properties, and the grading of evidence are presented in Table 7. No competing interests were disclosed in any of the included papers.

**Table 7:** Quality of studies on measurement properties and methodological rating of the instruments

Scale	Structural validity	Internal consistency	Cross-cultural validity/ measurement invariance	Reliability
**Climate Change Health Literacy Scale (CCHLS)**				
Risk of Bias	Doubtful	Very good	NA	NA
Criteria for good measurement properties	Sufficient (+)	Sufficient (+)	NA	NA
Quality of evidence	Low (due to RoB)	High	-	-
**Sustainable Food Literacy Scale**				
Risk of Bias	Very good	Very good	Very good	NA
Criteria for good measurement properties	Sufficient (+)	Sufficient (+)	Sufficient (+)	NA
Quality of evidence	High	High	High	-
**Sustainable Food Literacy Scale – Turkish Version**				
Risk of Bias	Doubtful	Doubtful	Doubtful	Doubtful
Criteria for good measurement properties	Sufficient (+)	Sufficient (+)	Indeterminate (?)	Insufficient (-)
Quality of evidence	Low (due to RoB)	Low (due to RoB)	-	Low (due to RoB)
**Climate Change and Infectious Disease Literacy Scale - Climate**				
Risk of Bias	Adequate	Very good	NA	NA
Criteria for good measurement properties	Sufficient (+)	Insufficient (-)	NA	NA
Quality of evidence	Moderate (due to RoB)	High	-	-
**Climate Change and Infectious Disease Literacy Scale - VBD**				
Risk of Bias	Adequate	Very good	NA	NA
Criteria for good measurement properties	Sufficient (+)	Insufficient (-)	NA	NA
Quality of evidence	Moderate (due to RoB)	High	-	-

RoB Risk of Bias; NA not applicable/no information available

The CCHLS [46] demonstrated high explained variance (67%) in exploratory factor analysis (EFA) and strong fit indices in confirmatory factor analysis (CFA) (χ²/df = 2.31, comparative fit index (CFI) CFI = 0.94, root mean square error of approximation (RMSEA) RMSEA = 0.06, Tucker-Lewis index (TLI) TLI = 0.93, standardised root mean residuals (SRMR) SRMR = 0.04). The study used the same sample size (*n* = 318) for EFA and CFA which introduced a risk of overfitting and was considered as a methodological flaw resulting in a doubtful risk of bias rating. Its internal consistency (Cronbach’s α = 0.94 overall; 0.75–0.93 for subscales) was rated as sufficient with a very good risk of bias rating. It was concluded that evidence regarding the sufficient structural validity of the CCHLS was of low quality, while there was high quality evidence indicating sufficient internal consistency.

Structural validity, internal consistency, and measurement invariance in the original SFLS [47] received a very good risk of bias rating. Separate EFA (*n* = 301) and CFA samples (CFA *n* = 667) were used yielding high explained variance (66.91%) and sufficient fit indices in CFA (χ²/df = 2.667, RMSEA = 0.05, CFI = 0.964) as well as a sufficient internal consistency (α = 0.85–0.95). Regarding measurement invariance for gender no important differences in the differential item functioning or CFI (Delta CFI = 0.001, Delta TLI = 0). A high quality of evidence was determined for sufficient results in all assessed measurement properties for the SFLS by Teng & Chih (Table 7).

The Turkish version of the SFLS [49] supported a five-factor structure explaining 77% of total variance in its EFA similar to the four-factor structure of the original SFLS, yet item-level performance was not compared to the original scale, and raw item results were unreported, preventing independent verification of adaptation adequacy and limiting a sufficient cross-cultural validity rating per COSMIN criteria (see Table 2). In the COSMIN risk of bias box 3 (criterion 4), box 4 (criterion 4), box 6 (criterion 7) the manuscript’s methodological shortcomings in the reporting of results, including the absence of the information on the used items or their modifications, the omission of a discussion and limitations section, and the absence of raw data or appendix data, were adjudged “doubtful”, thereby conferring an overall “doubtful” rating upon structural validity, internal consistency, and reliability (see Additional file 3 for full analysis). It was therefore decided that the results of both SFLS cannot be summarised [17].

The Climate Change and Infectious Disease Literacy Scale [48] used a 60:40 split of the total sample (*n* = 300), giving a CFA sample size (*n* = 121) which was considered adequate for the number of 52 items in the risk of bias rating. EFA revealed low explained variance (37.2% for the climate subscale and 28.5% for the VBD subscale) in particular regarding behavioural items. Yet CFA fit indices met thresholds (RMSEA = 0.015 in climate subscale and RMSEA = 0.00 in VBD subscale). Thereby, findings indicated moderate-certainty evidence for sufficient structural validity and high quality of evidence about insufficient internal consistency (α = 0.45; α = 0.47) in both subscales.

In conclusion, the SFLS demonstrated sufficient structural validity, internal consistency, and measurement invariance supported by very good quality of evidence, while its Turkish version had low quality evidence for sufficient structural validity and internal consistency, as well as low certainty evidence of insufficient reliability. The CCHLS showed sufficient structural validity with low quality of evidence alongside high-quality evidence for sufficient internal consistency. The Climate Change and Infectious Disease Literacy Scale exhibited moderate quality evidence for sufficient structural validity but high certainty evidence of insufficient internal consistency.

## Discussion

With this systematic review, we identified four subscales of three instruments (CCHLS, SFLS, and two subscales of the Climate Change and Infectious Disease Literacy Scale). We found all instruments developed deductively with few information on selection criteria; none of the instruments reported a qualitative concept elicitation through target population interviews or focus groups. Pilot testing was limited to CCHLS [46], where the target population provided face-to-face comprehensibility feedback. Content validity was assessed in all studies quantitatively through expert panels focussing on relevance and lacking the assessment of comprehensiveness and comprehensibility across scales. Three instruments [46–48] demonstrate sufficient structural validity supported by very good or moderate quality of evidence. The SFLS [47] demonstrated sufficient results in the assessed measurement properties (structural validity, internal consistency, and measurement invariance), supported by very good quality of evidence in its original, while its Turkish adaptation [49] had low methodological quality in its assessed measurement properties and insufficient reliability. The CCHLS showed high-quality evidence for sufficient internal consistency, while the Climate Change and Infectious Disease Literacy Scale exhibited insufficient internal consistency.

### Heterogeneity of concepts and constructs

We encountered unavoidable heterogeneity in constructs and concepts across studies. While COSMIN allows for some flexibility in handling such heterogeneity, we maintained methodological rigour by consistently applying a detailed, pre-defined concept and construct description. This pattern, both in the limited number of studies and the observed heterogeneity, mirrors findings from previous reviews in the field [14, 16, 24, 25], reinforcing the need for standardised terminology and transparent documentation in future research.

We observed that methodological studies from the health literacy research field have had limited influence on instrument development and construct understanding of the analysed scales. The prior scoping review [15] informing construct understanding of the Climate Change and Infectious Disease Literacy Scale [48] equated ‘literacy’ with “science,” “education,” and “knowledge” without further elaboration, representing an overly broad conceptualisation that lacks specificity regarding the targeted literacy dimensions. This observation aligns with a prior COSMIN analysis of climate “knowledge and awareness” instruments in nursing, which identified significant heterogeneity across eight retained measurement instruments employing varied terminology to define their constructs, including “attitudes, perceptions, environmental awareness, environmental sensitivity, environmental attitudes, behaviours, motivations, concerns, optimism, and experience” [16], omitting a health literacy focus.

In contrast, the work of Pleasant and McKinney emphasises that health literacy measurement must be explicitly grounded in a testable theory and construct [50].

### Gaps in description of instrument development

The authors consistently justified the relevance and rationale for assessing these constructs. Nayir and colleagues observed a high number of scales regarding general climate change attitudes, which in the their view “do not adequately address how these factors influence health-related behaviours and resilience” [46]. Matlack and colleagues describe the objective of the assessment of the construct as strengthening the target groups’ climate change literacy to “allow them to make better informed personal and community health decisions to reduce their climate impact and undertake adaptive behaviors” [48]. Yet the development processes across all scales lacked theoretical frameworks and details regarding construct specification and item‑generation procedures.

The studies for all instruments emphasised the reporting of primarily quantitative analyses, while we observed little focus on the development and content validity assessments in all four scales. Measurement objectives were vaguely described. This contrasts with COSMIN standards, which consider content validity as the most important measurement property, as the instrument’s items must adequately reflect the construct of interest [18]. For adequately rating the content validity, a clearly documented development process, including specification of the construct, context, and target population to be measured, is required [19]. Missing information for the characteristics of the included instruments (Table 2), including language and scoring algorithm, was provided by the authors via e-mail.

Given the uncertainty regarding the qualitative concept elicitation process and the unclear origins of the constructs, we sought clarification from all authors. Teng and Chih indicated that the scale was informed by an earlier exploratory interview study involving 27 household food providers [51] conducted prior to scale development, which was neither reported nor cited in their publication [47]. This foundational study could not be included in the current analysis, as it did not cover the construct “sustainable food literacy” nor meet the criteria for a development study.

These findings align with similar observations in other COSMIN systematic reviews: Gärtner and colleagues critically noted that published studies often lack essential details on constructs being measured, with the majority of their 40 reviewed instruments on shared decision-making lacking transparency in instrument development studies, with qualitative concept elicitation often missing or methodologically poor [52]. They emphasised the relevance of a well-defined construct definition and thorough content validity evaluation as critical initial stages in the development and validation of measurement instruments [52].

### Implications from the critical review of excluded studies

The limited number of instruments and validation studies reflects the field’s nascent stage. While formally excluded, the analysis of near-eligible studies confirmed the need for development and validation studies. In the context of Planetary Health Literacy, we found two pre- and post-course surveys to evaluate the effectiveness of transformative teaching in paediatrics and in nutrition [31, 32]. Neither reported a formal development or validation process, despite being considered potentially suitable for future research. Furthermore, it was found that the conceptual foundation in the Nurses’ Environmental Awareness Tool (NEAT) [41, 45], while being well-developed and validated for addressing nurses’ awareness, knowledge of environmental issues, and behaviours at home and work, did not integrate the environment-health nexus, systemic interdependencies, and transformative changes necessary for a Planetary or One Health approach, resulting in exclusion. The Climate, Health, and Nursing Tool (CHANT) [34, 35], a validated and standardised instrument, is grounded in the I-Change model, which posits that attitudes, self-efficacy, and social influences drive motivation and behaviour [34, 35], and aligns conceptually with the environment-health interdependency. Yet it does not capture health literacy or similar competency domains, limiting its inclusion due to the construct to be measured.

### Utilisation and applications of included scales in subsequent research

Of the instruments included, the SFLS is the only one that has been further adapted and used across diverse contexts. Teng and Chih emphasised the evaluative purpose of the SFLS as demonstrated in a recent assessment of curriculum effectiveness [53]. Despite its low evidence for sufficient validation, the Turkish adaptation of the SFLS [49] has been widely applied, e.g., to explore the relationship between sustainable food literacy and water footprint behaviour [54] or organic food consumption [55] as well as to understand dietary behaviours: While Özata-Uyar et al. [56] and Eroğlu et al. [57] suggest a correlation between sustainable food literacy and Mediterranean diet adherence in Turkish young adults, studies from Helvacı et al. [58] and Irgat et al. [59] found only weak correlations.

### Research gaps and implications for research

This systematic review identified several critical research gaps regarding the measurement of Planetary and One Health Literacy. First, we found the included instruments to rely exclusively on single-point, cross-sectional self-reported measures. Future research should consider incorporating objective, performance-based assessments. Second, this review found a notable lack of transparency in the development process of existing measurement instruments. Future instrument development must follow a clear, theory-driven approach which is fully documented in manuscripts or appendices. This includes grounding the instrument in established frameworks or using structured qualitative methods in concept elicitation studies with experts and target groups to identify core constructs and domains. Third, the included studies solely relied on expert feedback. Future instrument development in the field of Planetary and One Health Literacy should follow participatory research to co-design instruments with the target population and vulnerable groups to adequately reflect principles of equity and justice. This would align with the Ophelia principles [60].

### Limitations

The findings of this systematic review should be interpreted considering the following limitations. While efforts were made to comprehensively identify relevant measurement instrument across multiple databases and reference lists, some studies, particularly those published in non-peer-reviewed journals, or as conference abstracts, may not have been covered. As such, the findings should be interpreted with awareness of this potential gap regarding included instruments.

The COSMIN methodology, while providing essential rigour for evaluating measurement properties, employs inherently stringent criteria. This approach can result in low overall ratings for instruments, which may not fully capture their potential utility or quality in practical settings [61]. Further challenges occurred, particularly when authors’ terminology and analytical approaches diverged from COSMIN’s definitions. For example, the authors of CCHLS and SFLS reported assessing discriminative validity through analyses, such as Average Variance Extracted or Average Shared Square Variance, which, according to COSMIN, fall under internal structural validity rather than construct validity. Additionally, limited information on the item development process hindered clear differentiation between instrument development phases and content validity assessment: While all studies involved experts via online forms, it was not reported whether they evaluated pilot or final versions of the instruments, or whether experts contributed new items or modified existing ones. Despite the use of expert input in the early development stages of all instruments, we applied a uniform approach by treating expert involvement as a content validity assessment due to the availability of quantitative data.

Following the COSMIN methodology explicitly recommends the COSMIN-adjusted GRADE approach in the final step to rate the certainty of evidence for measurement properties. However, the standard GRADE approach is typically applied to accumulated evidence, whereas our review mainly relied on single and context-dependent studies. The COSMIN-adapted GRADE approach accounts for these constraints by downgrading the certainty of evidence accordingly.

## Conclusion

This review serves as an overview for researchers and practitioners to identify the most suitable measurement instruments tailored to their requirements. We did not find any fully validated instrument for the measurement of Planetary and One Health Literacy. The study demonstrates considerable variability in concepts and constructs. The four included subscales examined primarily the construct of knowledge, attitudes, and behaviours with regard to climate health literacy, as well as sustainable food literacy. We thereby recommend greater integration of health literacy domains and perspectives into instrument development research, providing valuable insights for both research areas.

Notably, the majority of existing studies have prioritised reporting in quantitative statistical validation over the actual conceptual development of the questionnaires themselves. As a result, the methodological quality in the instrument development phase has been consistently weak, as reflected in the low or very low quality of evidence for content validity. This highlights the critical importance of clearly articulating the intended context of use and target population prior to selecting items or designing any instrument. Furthermore, research efforts should place stronger emphasis on engaging the intended user population throughout the development process. Although the COSMIN checklist is known for its stringent assessment criteria, the findings underscore a pressing need for substantial improvements in designing measurement instruments.

## Supplementary Information

Below is the link to the electronic supplementary material.

Supplementary Material 1: Additional file 1 (.docx): Search term. Includes the full search string in different databases and the structural decomposition of search terms as well as the documentation of records per database

Supplementary Material 2: Additional file 2 (.docx): Databases specific for outcome measurement instruments. Includes the names and website links of the searched databases specific for outcome measurement instruments

Supplementary Material 3: Additional file 3 (.xlsx): COSMIN review management file. This Excel file contains all extracted data (e.g., instrument characteristics) and analyses (e.g., psychometric properties, risk of bias) of included instruments

Supplementary Material 4: Additional file 4 (.xlsx): Data extraction of excluded articles with reasons for exclusion

## Data Availability

The dataset supporting the conclusions of this article is included within the article and its additional files.

## Abbreviations

COSMIN: Consensus–based Standards for the Selection of Health Measurement Instruments
PROMs: Patient–Reported Outcome Measures
AUC: Area Under the Curve
CCHLS: Climate Change Health Literacy Scale
CFA: Confirmatory Factor Analysis
CFI: Comparative Fit Index
CTT: Classical Test Theory
DIF: Differential Item Functioning
EFA: Exploratory Factor Analysis
ICC: Intraclass Correlation Coefficient
IRT: Item Response Theory
LoA: Limits of Agreement
MIC: Minimal Important Change
RMSEA: Root Mean Square Error of Approximation
SDC: Smallest Detectable Change
SEM: Standard Error of Measurement
SFLS: Sustainable Food Literacy Scale
SRMR: Standardized Root Mean Square Residuals
TLI: Tucker–Lewis Index
VBD: Vector–borne diseases
