# Supplementary material for: Quality of measurement instruments assessing Planetary and One Health Literacy related constructs: a systematic review
Source: Health Qual Life Outcomes. 2026 Oct 16;24:132. doi: 10.1186/s12955-026-02652-3 (PMC13675952; doi:10.1186/s12955-026-02652-3)
Supplement: Supplementary file 1 [file 12955_2026_2652_MOESM1_ESM.docx]

# Additional file 1: Search term

##### Table 1: Structural decomposition of search terms

| Categories | *Related search terms* |
| --- | --- |
| **Construct of interest** | health AND (literacy or competenc*) |
| **Concept of interest** | “eco literacy” OR “planetary health” OR “one health” OR eco-health OR eco$health OR eco-medic* OR eco$medic* OR climate* OR biodiversity* OR "environmental sustainability" |
| **Instrument (type)***  *adapted from Terwee et al. 2009 [22] | questionnaire* OR ((scal* OR instrument) NEAR/4 develop*) OR instrumentation OR validation OR psychometr* OR clinimetr* OR clinometr* OR assessment OR "observer variation" OR "reproducibility of results" OR reproducib* OR "discriminant analysis" OR reliab* OR unreliab* OR "coefficient of variation" OR "internal consistency" OR (cronbach* AND (alpha OR alphas)) OR (item AND (correlation* OR selection* OR reduction*)) OR "precise values" OR test-retest OR (test AND retest) OR (reliab* AND (test OR retest)) OR ((replicab* OR repeated) AND (measure OR measures OR findings OR result OR results OR test OR tests)) OR discriminative OR "factor analysis" OR "factor analyses" OR "factor structure" OR "factor structures" OR subscale* OR (multitrait AND scaling AND (analysis OR analyses)) OR "item discriminant" OR "interscale correlation" OR "standard error of measurement" OR "Item response model" OR IRT OR Rasch OR "Differential item functioning" OR DIF OR "computer adaptive testing" OR "item bank" |

##### Table 2: Search strategy and result documentation across databases

| **Database** | **Search String** | **Number of Results** |
| --- | --- | --- |
| Web of Science Core Collection | TS=(health AND (((literacy or competenc*) NEAR/4 (“planetary health” OR climate* OR biodiversity* OR one$health OR “one health” OR eco-health OR eco$health OR eco-medic* OR eco$medic*)) OR “eco literacy” OR ("environmental sustainability" AND (literacy OR competenc*))) AND (questionnaire* OR ((scal* OR instrument) NEAR/4 develop*) OR instrumentation OR validation OR psychometr* OR clinimetr* OR clinometr* OR assessment OR "observer variation" OR "reproducibility of results" OR reproducib* OR "discriminant analysis" OR reliab* OR unreliab* OR "coefficient of variation" OR "internal consistency" OR (cronbach* AND (alpha OR alphas)) OR (item AND (correlation* OR selection* OR reduction*)) OR "precise values" OR test-retest OR (test AND retest) OR (reliab* AND (test OR retest)) OR ((replicab* OR repeated) AND (measure OR measures OR findings OR result OR results OR test OR tests)) OR discriminative OR "factor analysis" OR "factor analyses" OR "factor structure" OR "factor structures" OR subscale* OR (multitrait AND scaling AND (analysis OR analyses)) OR "item discriminant" OR "interscale correlation" OR "standard error of measurement" OR "Item response model" OR IRT OR Rasch OR "Differential item functioning" OR DIF OR "computer adaptive testing" OR "item bank")) | 98 |
| CINAHL, PsycInfo, Psyndex, Greenfile  via EBSCO | (health AND (((literacy or competenc*) N4 (“planetary health” OR climate* OR biodiversity* OR one#health OR “one health” OR eco-health OR eco#health OR eco-medic* OR eco#medic*)) OR “eco literacy” OR ("environmental sustainability" AND (literacy OR competenc*))) AND  (questionnaire* OR ((scal* OR instrument) N4 develop*) OR instrumentation OR validation OR psychometr* OR clinimetr* OR clinometr* OR assessment OR "observer variation" OR "reproducibility of results" OR reproducib* OR "discriminant analysis" OR reliab* OR unreliab* OR "coefficient of variation" OR "internal consistency" OR (cronbach* AND (alpha OR alphas)) OR (item AND (correlation* OR selection* OR reduction*)) OR "precise values" OR test-retest OR (test AND retest) OR (reliab* AND (test OR retest)) OR ((replicab* OR repeated) AND (measure OR measures OR findings OR result OR results OR test OR tests)) OR discriminative OR "factor analysis" OR "factor analyses" OR "factor structure" OR "factor structures" OR subscale* OR (multitrait AND scaling AND (analysis OR analyses)) OR "item discriminant" OR "interscale correlation" OR "standard error of measurement" OR "Item response model" OR IRT OR Rasch OR "Differential item functioning" OR DIF OR "computer adaptive testing" OR "item bank")) | 31 (only TI/AB) |
| Scopus | TITLE-ABS ( health ) AND TITLE-ABS (((literacy or competenc*) W/4 (“planetary health” OR climate* OR biodiversity* OR one?health OR “one health” OR eco-health OR eco?health OR eco-medic* OR eco?medic*)) OR “eco literacy” OR ("environmental sustainability" AND (literacy OR competenc*))) AND TITLE-ABS-KEY (questionnaire* OR ((scal* OR instrument) W/4 develop*) OR instrumentation OR validation OR psychometr* OR clinimetr* OR clinometr* OR assessment OR "observer variation" OR "reproducibility of results" OR reproducib* OR "discriminant analysis" OR reliab* OR unreliab* OR "coefficient of variation" OR "internal consistency" OR (cronbach* AND (alpha OR alphas)) OR (item AND (correlation* OR selection* OR reduction*)) OR "precise values" OR test-retest OR (test AND retest) OR (reliab* AND (test OR retest)) OR ((replicab* OR repeated) AND (measure OR measures OR findings OR result OR results OR test OR tests)) OR discriminative OR "factor analysis" OR "factor analyses" OR "factor structure" OR "factor structures" OR subscale* OR (multitrait AND scaling AND (analysis OR analyses)) OR "item discriminant" OR "interscale correlation" OR "standard error of measurement" OR "Item response model" OR IRT OR Rasch OR "Differential item functioning" OR DIF OR "computer adaptive testing" OR "item bank") | 106 |
| Ovid (Embase und Medline)* | (health AND (((literacy or competenc*) adj4 (“planetary health” OR climate* OR biodiversity* OR one$health OR “one health” OR eco-health OR eco$health OR eco-medic* OR eco$medic*)) OR “eco literacy” OR ("environmental sustainability" AND (literacy OR competenc*))) AND (questionnaire* OR ((scal* OR instrument) adj4 develop*) OR instrumentation OR validation OR psychometr* OR clinimetr* OR clinometr* OR assessment OR "observer variation" OR "reproducibility of results" OR reproducib* OR "discriminant analysis" OR reliab* OR unreliab* OR "coefficient of variation" OR "internal consistency" OR (cronbach* AND (alpha OR alphas)) OR (item AND (correlation* OR selection* OR reduction*)) OR "precise values" OR test-retest OR (test AND retest) OR (reliab* AND (test OR retest)) OR ((replicab* OR repeated) AND (measure OR measures OR findings OR result OR results OR test OR tests)) OR discriminative OR "factor analysis" OR "factor analyses" OR "factor structure" OR "factor structures" OR subscale* OR (multitrait AND scaling AND (analysis OR analyses)) OR "item discriminant" OR "interscale correlation" OR "standard error of measurement" OR "Item response model" OR IRT OR Rasch OR "Differential item functioning" OR DIF OR "computer adaptive testing" OR "item bank")).mp. | 144 |
