# Supplementary material for: Quality of measurement instruments assessing Planetary and One Health Literacy related constructs: a systematic review
Source: Health Qual Life Outcomes. 2026 Oct 16;24:132. doi: 10.1186/s12955-026-02652-3 (PMC13675952; doi:10.1186/s12955-026-02652-3)
Supplement: Supplementary file 2 [file 12955_2026_2652_MOESM2_ESM.docx]

**Additional File 2: Databases specific for outcome measurement instruments**

Searched databases (using the terms ‘Climate’, ‘Planetary Health’, or ‘One Health’):

- PROQOLID (<https://eprovide.mapi-trust.org/about/about-proqolid>)
- HealthMeasures (<https://www.healthmeasures.net/>)
- NIHR CRD Database (<https://www.crd.york.ac.uk/CRDWeb/HomePage.asp>)
- the COSMIN database of systematic reviews of outcome measurement instruments (<http://www.cosmin.nl/database-of-systematic-reviews.html>)
- the Test Archive of Leibniz Institute for Psychology Information (<https://www.testarchiv.eu/>)
- PubPsych search engine (<https://pubpsych.zpid.de/pubpsych/>)

Yielding 1 additional record for the screening.
